# Strontium Peroxide-Loaded Composite Scaffolds Capable of Generating Oxygen and Modulating Behaviors of Osteoblasts and Osteoclasts

**DOI:** 10.3390/ijms23116322

**Published:** 2022-06-05

**Authors:** Sheng-Ju Lin, Chieh-Cheng Huang

**Affiliations:** Institute of Biomedical Engineering, National Tsing Hua University, Hsinchu 30013, Taiwan; andrew5758@gapp.nthu.edu.tw

**Keywords:** strontium, oxygen-generating biomaterials, PLGA, composite scaffold

## Abstract

The reconstruction of bone defects remains challenging. The utilization of bone autografts, although quite promising, is limited by several drawbacks, especially substantial donor site complications. Recently, strontium (Sr), a bioactive trace element with excellent osteoinductive, osteoconductive, and pro-angiogenic properties, has emerged as a potential therapeutic agent for bone repair. Herein, a strontium peroxide (SrO_2_)-loaded poly(lactic-*co*-glycolic acid) (PLGA)-gelatin scaffold system was developed as an implantable bone substitute. Gelatin sponges serve as porous osteoconductive scaffolds, while PLGA not only reinforces the mechanical strength of the gelatin but also controls the rate of water infiltration. The encapsulated SrO_2_ can release Sr^2+^ in a sustained manner upon exposure to water, thus effectively stimulating the proliferation of osteoblasts and suppressing the formation of osteoclasts. Moreover, SrO_2_ can generate hydrogen peroxide and subsequent oxygen molecules to increase local oxygen tension, an essential niche factor for osteogenesis. Collectively, the developed SrO_2_-loaded composite scaffold shows promise as a multifunctional bioactive bone graft for bone tissue engineering.

## 1. Introduction

The reconstruction of bone defects remains challenging. To repair the defect site, surgeons commonly harvest autogenous bone tissues that possess significant osteoinductive, osteoconductive, and angiogenic potentials; these tissues are the gold standard clinical graft material for bone regeneration [1,2,3,4]. However, the utilization of bone autografts remains limited by several drawbacks, such as the creation of secondary surgical sites, limited availability of graft tissue, and substantial donor site complications [2,5]. To address these issues, researchers have made major efforts to develop biomaterial- and composite-based bone grafts [6,7,8,9,10,11,12]. As native bone tissue is mainly composed of mineralized collagen fibrils, collagen and its derivatives have been extensively investigated as bone substitutes owing to their excellent biocompatibility and biodegradability [13]. Nevertheless, these collagen-based grafts are unfortunately still far from ideal due to their inferior mechanical strength and limited ability to promote bone regeneration [6,14,15], thus necessitating the development of new grafting materials.

Recently, strontium (Sr), a bioactive trace element in the human body, has emerged as a potential therapeutic owing to its dual role in regulating bone metabolism: enhancing bone formation by stimulating osteoblasts and suppressing bone resorption by inhibiting osteoclasts [16,17,18,19,20,21]. For example, strontium ranelate has been utilized as a therapeutic agent to treat postmenopausal osteoporosis [20,22]. Sr-containing composites have also been developed as efficient bone graft materials [16,18]. Moreover, studies have demonstrated that Sr can induce angiogenesis [23,24], which is essential for bone regeneration. Although these Sr-modified bone substitutes exhibit enhanced osteoinductive, osteoconductive, and pro-angiogenic properties, trauma- or surgery-induced vascular disruption may lead to severe tissue hypoxia, delayed osteoblast differentiation, or even cell death, thus impairing the ultimate efficiency of bone regeneration [25,26]. Hyperbaric oxygen therapy, which involves administrating oxygen in a pressurized chamber, is a clinically available strategy for increasing tissue oxygen tension [25,27]. Without intact vasculature, however, the improvement of oxygenation in bone defect sites could be limited [28]. Moreover, patients cannot be exposed to hyperbaric conditions for a prolonged period [28]. Therefore, engineering bone grafts with a sustained oxygen evolving capacity to improve tissue oxygenation may efficiently promote bone repair.

Recently, calcium peroxide (CaO_2_)-based oxygen-generating biomaterials have been designed to improve local oxygen tension and support cell survival under hypoxic conditions [29,30,31,32,33,34]. The peroxide particles can react with water molecules, thus generating hydrogen peroxide (H_2_O_2_) that can be decomposed into oxygen by using suitable catalysts [28,33]. In one of our previous works, manganese dioxide (MnO_2_) was used as the catalyst, and both CaO_2_ and MnO_2_ powders were encapsulated into poly(lactic-*co*-glycolic acid) (PLGA) microparticles to control the reaction rate by limiting water infiltration [32]. The thus-developed injectable microparticles can serve as depots to release oxygen in a sustained manner and relieve cellular hypoxia [32]. Without suitable porous scaffold architecture and osteoconductivity, however, these microparticles may not effectively serve as ideal bone grafting materials.

Herein, a multifunctional strontium peroxide (SrO_2_)-based oxygen-generating scaffold was developed as an implantable bone substitute. Similar to CaO_2_, SrO_2_ can generate H_2_O_2_ and the subsequent oxygen upon exposure to water. Moreover, we anticipate that the release of Sr^2+^ could effectively enhance new bone formation and inhibit bone resorption. To our knowledge, this is the first report of developing a strontium-based biomaterial with oxygen evolution capacity. To fabricate the proposed multifunctional implantable constructs, we immobilized SrO_2_ and MnO_2_ powders on gelatin sponges using PLGA. The gelatin sponge serves as a porous osteoconductive scaffold, while PLGA not only reinforces the mechanical strength of the gelatin but also controls the rate of oxygen generation by limiting water infiltration. We anticipate that the developed SrO_2_-encapsulated oxygen-generating scaffolds can be used to promote the formation of new bone tissues.

## 2. Materials and Methods

### 2.1. Materials

PLGA with a lactide:glycolide molar ratio of 75:25 and an inherent viscosity of 0.53 dL/g was purchased from Green Square Material (Taipei, Taiwan). Gelatin sponges (Spongostan^TM^; MS0001) was acquired from Ferrosan Medical Devices (Søborg, Denmark). SrO_2_, MnO_2_, dichloromethane (DCM), and acid phosphatase staining kits were purchased from Sigma-Aldrich (St. Louis, MO, USA). Mouse MC3T3-E1 preosteoblasts and RAW 264.7 macrophages were obtained from the Bioresource Collection and Research Center, Food Industry Research and Development Institute (Hsinchu, Taiwan). Cell culture reagents were purchased from Thermo Fisher Scientific (Waltham, MA, USA). Receptor activator of nuclear factor κB ligand (RANKL) was acquired from Peprotech (Rocky Hill, NJ, USA). All other chemicals and reagents used were of analytical grade.

### 2.2. Preparation of SrO_2_ + MnO_2_@PLGA/Gelatin Scaffolds

PLGA solution was prepared by dissolving powder in DCM. After combination with SrO_2_ and MnO_2_ powder, 100 µL of the acquired solution was transferred onto each piece of the gelatin sponge with a volume of 5 × 5 × 0.5-mm^3^. The samples were incubated in a hood overnight for solvent evaporation, and the resultant SrO_2_ + MnO_2_@PLGA/gelatin scaffolds were collected for further use. The scaffolds were observed by scanning electron microscopy (SEM; JSM-7610-F; JEOL, Tokyo, Japan).

### 2.3. Oxygen and Sr^2+^ Release Profile of SrO_2_ + MnO_2_@PLGA/Gelatin Scaffolds

Two SrO_2_ + MnO_2_@PLGA/gelatin scaffolds were transferred into 5 mL of deoxygenated phosphate-buffered saline (PBS). The dissolved oxygen concentrations were measured with an InLab OptiOx DO sensor (Mettler Toledo, Greifensee, Switzerland) [32,35]. Furthermore, the pH value of the solution was detected with a pH meter (ST3100; OHAUS, Parsippany, NJ, USA). For determination of the release profile of Sr*^2+^*, the PBS solutions were analyzed with inductively coupled plasma-mass spectrometry (ICP-MS; Agilent 7500ce; Agilent, Santa Clara, CA, USA) [32].

### 2.4. Cell Culture

MC3T3-E1 preosteoblasts and RAW 264.7 macrophages were purchased from the Bioresource Collection and Research Center, Food Industry Research and Development Institute, Hsinchu, Taiwan. MC3T3-E1 cells were maintained in ascorbic acid-free α minimum essential medium containing 10% fetal bovine serum (FBS; GE Healthcare Bio-Sciences, Pittsburgh, PA, USA), 100 U/mL penicillin, and 100 μg/mL streptomycin [32]. RAW 264.7 cells were cultured in Dulbecco’s modified minimum essential medium (DMEM) supplemented with 10% FBS, 2 mM glutamine, 100 U/mL penicillin, and 100 μg/mL streptomycin [36].

### 2.5. Biocompatibility of SrO_2_ + MnO_2_@PLGA/Gelatin Scaffolds

MC3T3-E1 cells were seeded in 48-well plates at a density of 2.5 × 10^4^ cells per well and incubated for 24 h before treatment with five SrO_2_ + MnO_2_@PLGA/gelatin scaffolds for 24 h. A Cell Counting Kit-8 assay (CCK-8; IMT Formosa New Materials, Kaohsiung, Taiwan) was used to quantify cell viability [37]. Wells that contained only culture medium were used as blank wells. Alternatively, MC3T3-E1 cells were inoculated directly on the surface of SrO_2_ + MnO_2_@PLGA/gelatin to assess their potential in serving as scaffolds. After a seven-day incubation, the scaffolds were fixed using 4% paraformaldehyde, stained with 4’,6-diamidino-2-phenylindole (DAPI) and Alexa Fluor 488-conjugated phalloidin (dilution ratio of 1:1000; Cat. No. A12379; Thermo Fisher Scientific) for visualization of nuclei and F-actin, respectively, and observed using a confocal laser scanning microscope (Carl Zeiss). The 3D rendering of the acquired fluorescent images was conducted using ZEN Blue software (Carl Zeiss) [38].

### 2.6. Osteoclast Differentiation

For the induction of osteoclast differentiation, RAW 264.7 cells were treated with 50 ng/mL RANKL with or without the SrO_2_ + MnO_2_@PLGA/gelatin scaffolds for five days [39]. The gene expression levels of *Trap* and *Mmp9* were determined using real-time quantitative polymerase chain reaction (qPCR) following MIQE guidelines [40]. Total RNA of the test cells was extracted using TRIzol reagent and reverse-transcribed into complementary DNA with a High Capacity Reverse Transcription Kit. Real-time qPCR was conducted with Power SYBR Green PCR Master Mix in the StepOnePlus Real-Time PCR System (Thermo Fisher Scientific) [41,42]. The primer sequences were as follows: *Gapdh* forward 5′-CTGCCACCCAGAAGACTGTG-3′ and reverse 5′-GGTCCTCAGTGTAGCCCAAG-3′ [43]; *Mmp9* forward 5′-GAAGGCAAACCCTGTGTGTT-3′ and reverse 5′-AGAGTACTGCTTGCCCAGGA-3′ [44]; *Trap* forward 5′-TCCTGGCTCAAAAAGCAGTT-3′ and reverse 5′-ACATAGCCCACACCGTTCTC-3′ [45]. The relative mRNA expression levels of the target genes were quantified and normalized to the expression of the housekeeping gene *Gapdh*. Furthermore, the experimental samples were stained with an acid phosphatase kit (Cat. No. 387A; Sigma-Aldrich) to detect the activity of tartrate-resistant acid phosphatase (TRAP) according to the manufacturer’s instructions.

### 2.7. Statistical Analysis

Statistical analyses were conducted using GraphPad Prism software (version 9.1; San Diego, CA, USA). All data are presented as the mean ± standard deviation. A two-tailed Student’s *t* test was used for comparisons between two groups. One-way analysis of variance (ANOVA) with Bonferroni correction was used for comparisons among groups. A *p* value of less than 0.05 was considered significant.

## 3. Results and Discussion

### 3.1. SrO_2_ + MnO_2_@PLGA/Gelatin Scaffolds Releases Oxygen and Strontium Ion

**Figure 1:**
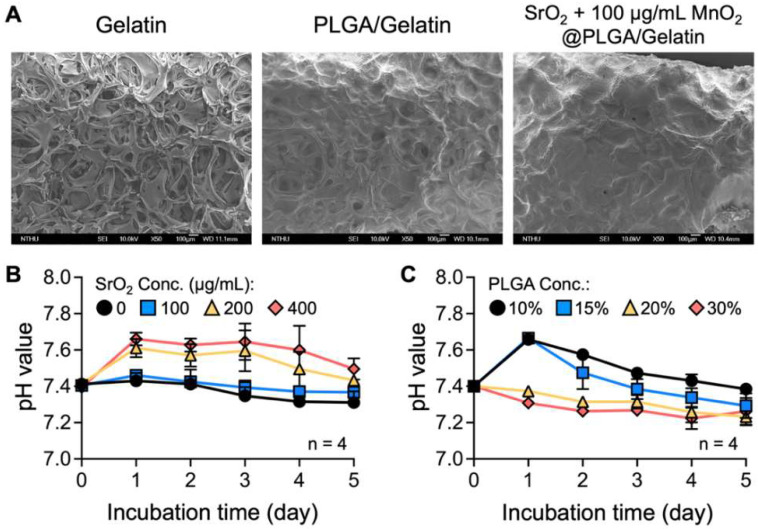
Fabrication and optimization of the SrO_2_ + MnO_2_@PLGA/gelatin scaffolds. (**A**) SEM images of the surface of the gelatin sponge and the prepared scaffolds. (**B**,**C**) The pH values of phosphate-buffered saline (PBS) following incubation with the developed scaffolds prepared with various parameters (*n* = 4). Data are the mean ± s.d.

For preparation of the scaffolds, SrO_2_ and MnO_2_ powder was dispersed in PLGA solution and transferred onto a gelatin sponge. As revealed in the SEM images in Figure 1A, the porous structure of the gelatin sponge was filled by PLGA. We first optimized the amount of SrO_2_ loaded in the scaffolds. Solid SrO_2_ particles are known to react with water and generate H_2_O_2_ and strontium hydroxide (Sr(OH)_2_), which can result in an increase in the environmental pH value and thus may lead to harmful effects on the surrounding tissue [46]. To prevent alkaline-induced cytotoxicity, we monitored the changes in the pH value of the PBS solution that was used to incubate the SrO_2_ + MnO_2_@PLGA/gelatin scaffolds. When prepared using 20% (*wt*/*v*) PLGA, the scaffolds that contained 200 or 400 µg/mL SrO_2_ powder resulted in an elevation of the pH value (Figure 1B), suggesting the accumulation of strontium hydroxide. Conversely, the samples that contained 100 µg/mL SrO_2_ powder did not result in a significant increase in pH value (Figure 1B).

We next attempted to optimize the PLGA concentration for scaffold fabrication. As indicated in the profile of pH value in Figure 1C, the scaffolds that were prepared with 10% or 15% PLGA led to remarkable elevation of pH compared to that prepared with 20% or 30% PLGA, which can be attributed to their higher hydrophobicity. Compared with our previous publication, in which 15% PLGA was used to encapsulate CaO_2_ powder [32], the incorporation of a gelatin sponge in the present study appears to promote water infiltration. Therefore, PLGA at a higher concentration was required to effectively control the dissolution rate of SrO_2_. Although scaffolds fabricated with an increased PLGA content were expected to have enhanced mechanical properties, immunological responses and the foreign body reaction may also be elicited after scaffold implantation [47]. Based on the aforementioned results, scaffolds that were prepared using 20% PLGA and embedded 100 µg/mL SrO_2_ powder were used for subsequent studies.

**Figure 2:**
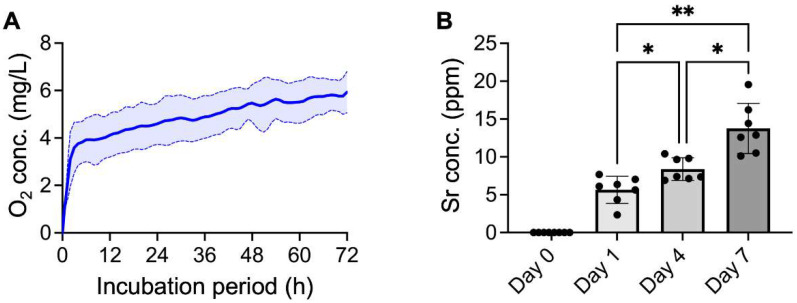
SrO_2_ + MnO_2_@PLGA/gelatin scaffolds release oxygen and strontium ions. The release kinetics of (**A**) oxygen (*n* = 4) and (**B**) strontium (*n* = 7) from the SrO_2_ + MnO_2_@PLGA/gelatin scaffolds incubated in PBS. * *p* < 0.05; ** *p* < 0.01. Data are the mean ± s.d.

In addition to Sr(OH)_2_, the dissolution of SrO_2_ also produces H_2_O_2_, which can be efficiently converted to oxygen in the presence of catalysts. As shown in the oxygen release profiles in Figure 2A, the concentration of dissolved oxygen in PBS solution increased gradually within the investigated period, suggesting that the SrO_2_ + MnO_2_@PLGA/gelatin scaffolds evolved the oxygen in a sustained manner. It was reported that direct use of solid peroxides, such as SrO_2_ and CaO_2_, in aqueous environments or tissues, leads to a burst release behavior of oxygen, which is considered to be harmful for the surrounding tissues [48]. Furthermore, the accompanying rapid exhaustion of peroxides indicates that only a short oxygen release period can be achieved [48]. In the present study, we successfully offset these adverse effects by tuning the PLGA/gelatin scaffolds, thus controlling the dissolution rate and the oxygen release behavior of SrO_2_. To determine the release kinetics of strontium, we collected the experimental PBS solution and analyzed it using ICP-MS. Similar to the release behavior of oxygen, the concentration of strontium increased gradually as time progressed, demonstrating the potential of the developed SrO_2_ + MnO_2_@PLGA/gelatin scaffolds to serve as an efficient depot for the sustained release of strontium (Figure 2B).

### 3.2. SrO_2_ + MnO_2_@PLGA/Gelatin Scaffolds Promote Proliferation of Preosteoblasts

**Figure 3:**
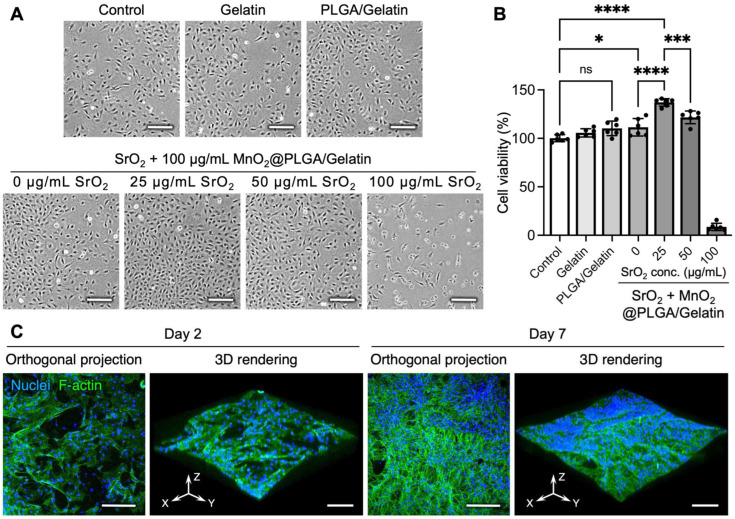
SrO_2_ + MnO_2_@PLGA/gelatin scaffolds promote the proliferation of preosteoblasts. (**A**) Representative phase contrast photomicrographs of MC3T3-E1 preosteoblasts that received various treatments and (**B**) the corresponding cell viability determined using a CCK-8 assay (*n* = 6). Scale bars, 200 µm. * *p* < 0.05; *** *p* < 0.005; **** *p* < 0.001; ns, not significant. Data are the mean ± s.d. (**C**) Representative orthogonal projection and 3D rendering of MC3T3-E1 cells grown on the scaffolds and incubated for two or seven days. Scale bars, 200 µm.

Next, to evaluate scaffold biocompatibility, we cultured MC3T3-E1 cells in the presence or absence of Transwell inserts that contained the prepared scaffolds. As revealed in the phase-contrast images and the corresponding results of the CCK-8 assay, cells that received plain gelatin sponges or PLGA/gelatin scaffolds exhibited comparable viability to that of the untreated cells (Figure 3A,B), suggesting that the compositions of the scaffolds did not affect the grown cells. However, treating cells with SrO_2_ + MnO_2_@PLGA/gelatin scaffolds that contained 25 µg/mL SrO_2_ resulted in a 37.1% increase in cell viability (*p* < 0.001 compared to the untreated control; Figure 3B). It has been reported that supplementation with Sr^2+^ can promote the proliferation of osteoblasts [9,19]. Hence, the Sr^2+^ released during SrO_2_ dissolution may enhance the proliferation of the MC3T3-E1 cells. Furthermore, although the bulk pH value was not changed significantly, the hydroxide ions released together with Sr^2+^ might establish mild local alkaline conditions, which have been reported to be beneficial for the proliferation of osteoblasts [49]. Nevertheless, as more SrO_2_ was encapsulated into the scaffold, the viability of the cells decreased dramatically (Figure 3A,B), probably owing to the accumulation of H_2_O_2_ and thus oxidative stress [27]. Although MnO_2_ was loaded into the scaffolds as a catalyst, if a high concentration of H_2_O_2_ is generated in a short period, the H_2_O_2_ molecules might diffuse out before being decomposed into oxygen and water [27]. As a result, the scaffolds that contained 25 µg/mL SrO_2_ without significant cytotoxicity were chosen for subsequent investigations.

To evaluate whether SrO_2_ + MnO_2_@PLGA/gelatin could serve as an efficient scaffold and provide physical support for preosteoblasts, we seeded MC3T3-E1 cells directly onto the surface of the prepared scaffolds. The confocal images indicated that the inoculated cells could adhere and proliferate on the surface of SrO_2_ + MnO_2_@PLGA/gelatin (Figure 3C), demonstrating its potential to function as a biocompatible scaffold system that can enhance the proliferation of preosteoblasts. Although PLGA is a synthetic polymer that has been extensively used as a tissue engineering scaffold, its cell adhesive capacity is considered to be not optimal [50,51]. Conversely, the potential of gelatin sponges to promote cell adhesion has been well documented, especially with osteoblasts [7,52]. By taking advantage of these materials, our data showed that the PLGA/gelatin composite scaffolds could be used as ideal tissue engineering scaffolds to support the adhesion and proliferation of the grown cells, which is in agreement with the literature [53,54,55].

### 3.3. SrO_2_ + MnO_2_@PLGA/Gelatin Scaffolds Inhibit Osteoclast Differentiation

**Figure 4:**
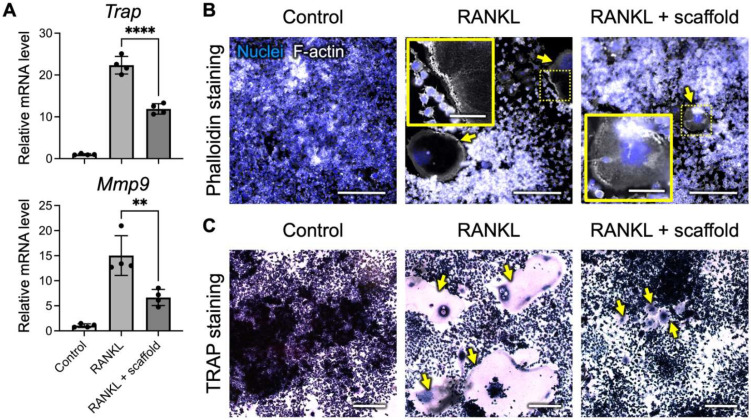
SrO_2_ + MnO_2_@PLGA/gelatin scaffolds inhibit osteoclast differentiation. (**A**) Expression levels of the osteoclast-specific genes *Trap* and *Mmp9* in the RAW 264.7 cells with or without receptor activator of nuclear factor κB ligand (RANKL) and scaffold treatment determined by quantitative PCR (*n* = 4). ** *p* < 0.01; **** *p* < 0.001. Data are the mean ± s.d. (**B**) Representative fluorescence images of F-actin staining and (**C**) TRAP staining of RAW 264.7 cells after receiving various treatments. Yellow arrows indicate the (**B**) actin ring or (**C**) TRAP-positive cells. The yellow boxes show the regions outlined by dotted lines with higher magnification. Scale bars, 200 µm. Scale bars in insert, 50 μm.

In addition to promoting osteogenesis, strontium was reported to inhibit bone resorption by suppressing the differentiation and maturation of osteoclasts [56,57,58]. Herein, murine RAW 264.7 macrophages were stimulated with RANKL to induce osteoclastogenesis in the presence or absence of SrO_2_ + MnO_2_@PLGA/gelatin scaffolds. As indicated by the qPCR results in Figure 4A, significantly enhanced expression of the *Trap* (22.3-fold increase vs. control; *p* < 0.001) and *Mmp9* genes (15.1-fold increase vs. control; *p* < 0.001) was detected in the RANKL-treated cells, suggesting their differentiation toward osteoclasts. Conversely, in the group that received the developed scaffolds, the mRNA levels of *Trap* and *Mmp9* exhibited 55.7% and 46.1% reductions, respectively, compared to those of the RANKL-stimulated group (Figure 4A; *p* < 0.01), suggesting that the SrO_2_ + MnO_2_@PLGA/gelatin scaffolds could effectively inhibit osteoclastogenesis.

As the formation of a thick-band actin ring in osteoclasts is required for their bone resorption activity [59,60], we next investigated whether the introduction of the SrO_2_ + MnO_2_@PLGA/gelatin scaffolds could affect the organization of the actin ring. As revealed by the fluorescence images of phalloidin staining in Figure 4B, RANKL treatment induced actin ring formation in RAW 264.7 cells. In the presence of the developed scaffolds, however, a disrupted actin ring was observed, suggesting the ability of the SrO_2_ + MnO_2_@PLGA/gelatin scaffold to suppress osteoclast activity. Furthermore, the formation of osteoclasts in the experimental samples was analyzed via TRAP staining. As revealed in Figure 4C, the number of TRAP-positive multinuclear cells in the RANKL-treated group increased significantly, while co-treatment with the scaffolds developed in the present study efficiently reduced the number of TRAP-positive cells. The abovementioned results demonstrated that the SrO_2_ + MnO_2_@PLGA/gelatin scaffolds could effectively inhibit osteoclastogenesis and reduce osteoclast activity and thus might suppress subsequent bone resorption.

Despite our success in fabricating composite scaffolds with the dual functions of oxygen and Sr^2+^ release, several limitations remain to be addressed prior to future translational applications. First, the present study only investigated the capacity of the SrO_2_ + MnO_2_@PLGA/gelatin scaffolds to modulate the behaviors of osteoblasts and osteoclasts under normal oxygen tension. It was reported that under hypoxic conditions (e.g., 1% oxygen concentration), the proliferation and differentiation potential of osteoblasts is remarkably inhibited [61,62]. Furthermore, the hypoxic niche contributes significantly to the activation of osteoclasts for bone resorption [63,64]. Therefore, further investigations of the synergistic effects of oxygen and Sr^2+^ released by the developed scaffold system are warranted in terms of promoting osteoblast proliferation/differentiation and suppressing osteoclast formation to further highlight the potential benefits of SrO_2_ + MnO_2_@PLGA/gelatin. Second, the present study only analyzed cellular behaviors in a short-term culture. As the release of oxygen and Sr^2+^ in a sustained manner by the developed SrO_2_ + MnO_2_@PLGA/gelatin has been verified, in vitro investigation with a prolonged cultivation period can help validate the advantages of incorporating a controlled release platform into the scaffold system. Furthermore, protein-based analyses of the effects of SrO_2_ + MnO_2_@PLGA/gelatin on osteogenesis and osteoclastogenesis are necessary to demonstrate the functionality of the scaffold system. Finally, animal investigations with a nonunion bone fracture model are needed to verify the in vivo therapeutic potential.

## 4. Conclusions

In summary, the present study demonstrated the potential of SrO_2_ + MnO_2_@PLGA/gelatin as a scaffold system for bone tissue engineering. By releasing oxygen and Sr^2+^, the developed scaffolds could increase local oxygen tension and modulate the behavior of osteoblasts and osteoclasts, respectively. Despite several limitations, the results obtained in the present study establish an important proof-of-concept for the future application of SrO_2_-based biomaterials for bone tissue engineering.

## Data Availability

Data are available from C.-C.H. upon request.
